# Comparing the genetic typing methods for effective surveillance and rabies control in Georgia

**DOI:** 10.3389/fmicb.2023.1243510

**Published:** 2023-12-01

**Authors:** Rene E. Condori, Natia Kartskhia, Lasha Avaliani, Marina Donduashvili, Tinatin Elbakidze, Ana Kapanadze, Emily G. Pieracci, Giorgi Maghlakelidze, Ashutosh Wadhwa, Clint N. Morgan, Mary Reynolds, Yu Li, Lena Ninidze

**Affiliations:** ^1^Poxvirus and Rabies Branch, Division of High Consequence Pathogens and Pathology, National Center for Emerging and Zoonotic Infectious Disease, Centers for Disease Control and Prevention, Atlanta, GA, United States; ^2^Veterinary Department, National Food Agency, Ministry of Environmental Protection and Agriculture, Tbilisi, Georgia; ^3^State Laboratory of Agriculture, Ministry of Environmental Protection and Agriculture, Tbilisi, Georgia; ^4^Center for Global Health, Centers for Disease Control and Prevention, Tbilisi, Georgia

**Keywords:** canine rabies, LN34 assay, genetic diversity, surveillance, rabies typing, molecular epidemiology

## Abstract

A full nucleoprotein gene sequencing of 68 isolates collected from passive rabies surveillance system in Georgia between 2015 and 2016 identified two distinct dog rabies phylogroups, GEO_V1 and GEO_V2, which both belonged to the cosmopolitan dog clade. GEO_V1 was found throughout the country and was further divided into four sub-phylogroups that overlapped geographically; GEO_V2 was found in the southeast region and was closely related to dog rabies in Azerbaijan. A sequence analysis of the full N gene, partial nucleoprotein gene of N-terminal and C-terminal, and the amplicon sequences of pan-lyssavirus RT-qPCR LN34 showed that all four sequencing approaches provided clear genetic typing results of canine rabies and could further differentiate GEO_V1 and GEO_V2. The phylogenetic analysis results vary and were affected by the length of the sequences used. Amplicon sequencing of the LN34 assay positive samples provided a rapid and cost-effective method for rabies genetic typing, which is important for improving rabies surveillance and canine rabies eradication globally.

## 1 Introduction

Rabies infection is recognized to be almost always deadly upon the appearance of clinical symptoms. The disease can be caused by any member of the *Lyssavirus* genus within the *Rhabdoviridae* family. *Lyssavirus* r*abies*, commonly known as rabies virus (RABV), is estimated to be responsible for at least 59,000 human rabies cases worldwide (Hampson et al., 2015). Rabies fatality is 100% preventable when appropriate post-exposure prophylaxis (PEP) is given on time (Kessels et al., 2019). The RABV genome encodes five proteins separated by intergenic regions: nucleoprotein (N), phosphoprotein (P), matrix protein (M), glycoprotein (G), and RNA-dependent RNA polymerase (L).

Most of the yearly estimated human deaths are caused by dogs, largely in the developing countries of Asia and Africa (Hampson et al., 2015), even though RABV transmission in dog populations is subject to mitigation. Rabies epizootics in wildlife species are particularly unpredictable, and early recognition of the virus in field samples is crucial to determining an outbreak or interspecific transmission (Fisher et al., 2018). Although dog rabies has been eliminated in most European countries, the risk of reintroduction persists (Kardamanidis et al., 2013; Ribadeau-Dumas et al., 2016). One important region that interconnects Europe and Asia is the Caucasus region, wherein the epidemiological cycle of canine rabies remains endemic (Zeynalova et al., 2015).

In Georgia, dog rabies is endemic and represents a serious threat to public health. Although the government implemented a rabies control program that helped to reduce the incidence of rabies in owned dogs, the disease re-emerged in the population of stray dogs and wild animals (Kartskhia et al., 2020). To control the disease across the country, a mass dog vaccination campaign in collaboration with local governments was established in 2013. Annually, the Georgian rabies control program vaccinates over 200,000 dogs against rabies. According to the data from the National Food Agency of the Ministry of Environmental Protection and Agriculture, between 2000 and 2017, 1,359 cases of rabies in dogs were detected. Between 2000 and 2014, at least 109 human rabies cases were recorded. No human cases were registered between 2015 and 2017, but three people died from rabies in 2018 and 2019; two occurred in the Russian-occupied territory of Abkhazia and one was associated with a jackal bite. A jackal infected with rabies was also detected in Tbilisi in 2016. Aside from what is known about the cosmopolitan dog RABV lineage that is circulating in Georgia, there is a lack of knowledge on the rabies reservoirs and its diversity as well as relevant information about other species playing a role as rabies reservoir hosts. In countries with limited resources, it may become challenging to accurately identify the reservoir host (Tiwari et al., 2021).

Although rabies is routinely tested using the Direct Fluorescent Antibody test (DFA), there is a gap in the knowledge of the molecular epidemiology of RABVs circulating in Georgia. In rabies-endemic countries, cattle are the second most affected species after dogs, although in some countries they are the most affected animal (Vos et al., 2014; Abdelmalik and Yahia, 2021). Although rabies in livestock is typically the end point of rabies transmission, rabies in cattle poses a potential risk for humans and has an impact on the local and national economy (Hampson et al., 2015). In settings where rabies cases are detected in domestic animals and wildlife, molecular tools may be useful to uncover the co-circulation of different rabies strains in the same region (Sadeuh-Mba et al., 2017).

The current standard for rabies testing is the DFA, which is recommended by the World Organization for Animal Health (2023) and is used as a primary diagnostic tool to confirm rabies in countries where dog rabies remains endemic, but the reliability of the test is tied to the quality of the reagents, the condition of the brain tissue, the quality of the fluorescent microscope, and the skills of the laboratory personnel (Gigante et al., 2018; Rupprecht et al., 2019). Alternately, the recently developed real-time RT-PCR LN34 assay, which is capable of detecting all known lyssavirus species in a single set-up, even if the sample is degraded or fixed in formalin, is capable of providing results in a short time but requires skilled personnel and validated reagents and PCR machines to guarantee reliable results (Wadhwa et al., 2017; Gigante et al., 2018). In addition, the amplicons of the LN34 assay can be subsequently sequenced to rapidly conduct the genetic typing (Condori et al., 2020). Although real-time amplicons are useful for rapid genetic typing, its resolution is not enough to conduct phylogenetic analysis; therefore, successive analyses using the partial or full N gene can still be necessary to understand rabies epidemiology. The N gene has been widely used to conduct phylogenetic analysis and differentiate rabies virus variants, particularly because this gene is highly conserved (Wunner et al., 1988).

With the aim of characterizing RABV strains circulating in Georgia and providing basic insights into the molecular epidemiology of rabies and its relationship with neighboring countries, a highly conserved non-coding 3′ leader region at the beginning of the rabies genome and subsequent full N genes were targeted for sequencing and analysis; also, a nucleotide comparison of the LN34 assay sequences was included as a rapid, cost-effective way of genetic typing.

## 2 Materials and methods

### 2.1 Animal samples

The dataset (*n* = 68) included samples collected by the rabies surveillance system in different administrative regions of Georgia. All samples were collected by the National Food Agency of Georgia during 2015–2016 and confirmed positive by the standard Direct Fluorescent Antibody (DFA) test at the State Laboratory of Agriculture in Georgia. As part of an ongoing collaborative effort to eliminate canine rabies in Georgia, aliquots of primary brain tissue were submitted to the U.S. Centers for Disease Control and Prevention (CDC) Poxvirus and Rabies Branch for genetic characterization.

### 2.2 Viral RNA purification, LN34 real time RT-PCR assay, and standard RT-PCR analysis

Following the manufacturer's recommendations, total RNA was extracted from approximately 100 mg of brain tissue using TRIzol reagent with Direct-zol RNA Miniprep kit (Zymo Research, Irvine, CA, USA). All RNA samples were tested by the LN34 Pan-lyssavirus real-time RT-PCR assay. Each sample was tested by duplicate, housekeeping gene β-actin that detects the host mRNA, and internal controls were included. To determine if the sample is positive, negative, or inconclusive, the cycle threshold (Ct) value ≤ 35 was considered for the LN34 assay and ≤ 33 for β-actin (Wadhwa et al., 2017; Gigante et al., 2018). To obtain the complete N gene, a conventional RT-PCR with overlapping primers was used (Condori-Condori et al., 2013).

### 2.3 Nucleotide sequencing and phylogenetic analysis

To obtain the complete N gene, purified PCR products were sequenced in an instrument 3,730 DNA analyzer using primers described previously (Condori-Condori et al., 2013). Nucleotide sequences were assembled and edited into Bioedit 7.0.5.3 software (Hall, 1999). The SADB19 (GenBank No. M31046) RABV strain was used as a reference.

Many Georgian samples had identical nucleotide sequences. To conduct the phylogenetic analysis from the identical samples, representative sequences were selected according to their location and species. A subset of 52 Georgian sequences generated in this study with 55 representative N gene sequences from neighboring countries and Europe were aligned using Clustal Omega on Geneious prime 2020.2.4 (https://www.geneious.com). The Bayesian Evolutionary Analysis by Sampling Tree (BEAST v1.10) (Suchard et al., 2018) software package was used to perform the phylogenetic analysis of the complete N gene. A General Time Reversible (GTR) substitution model with discrete gamma distribution of rate variation was recognized to fit better by the Bayesian Information Criterion (BIC) in jModelTest2.1.10 (Darriba et al., 2012). A Bayesian Markov Chain Monte Carlo (MCMC) analysis in BEAST was implemented using uncorrelated-relaxed and lognormal-relaxed clock models with constant size coalescent. The simulation was performed for 50 million generations with sampling every 5,000 states. The MCMC inferences were visualized using Tracer v1.7.1 (Rambaut et al., 2018). The maximum clade credibility (MCC) tree was combined by tree Annotator v1.10.4, and the consensus tree was edited and visualized using Fig Tree v1.4.0 (Rambaut, 2009). Complete N gene sequences generated in this study were deposited in GenBank with accession numbers ranging from MT079888 to MT079955, and the map with the sample location was generated using Microreact (Argimon et al., 2016). To observe if the tree topology changes, the entire N gene was trimmed with the N-terminal and C-terminal regions cut to 400 bp. Phylogenetic analysis of the partial N gene was conducted using the same parameters described for the complete N gene. An extended set of 124 sequences of the N-terminal and 107 sequences of the C-terminal were used to construct the phylogenetic tree.

To determine the potential benefit of the LN34 assay for rapidly determining the reservoir host of the RABV, products of the LN34 assay were purified using the MinElute PCR Purification Kit (Qiagen). In total, 10–20 ng of purified amplicons was added to the sequence reaction containing 2 μl of BigDye Terminator v1.1 Cycle sequencing kit (Applied Biosystems), 1.5 μl of sequencing buffer, and 2 μl of LN34 primer (3.2 μM) in a final volume of 20 μl. The following thermal cycling conditions were used: 96°C for 60 s and 25 cycles of 96°C for 10 s, 56°C for 5 s, and 60°C for 60 s. The amplicons were sequenced using the 3,730 DNA Analyzer with the fastSeq50_POP7 run module. The LN34 sequences (164 bp) were trimmed to 115 bp by removing the regions where the primers cleave. Representative sequences used for the complete and partial N gene analysis were included and trimmed to match the size of the sequences of the LN34. A subset of 44 sequences was aligned on Geneious prime.

## 3 Results

The 68 rabies-positive samples included in this study were collected during 2015 and 2016 by the passive rabies surveillance system in Georgia and forwarded to the U.S. CDC for genetic characterization as part of a joint government partnership. From the sequences generated at the U.S. CDC, 40 isolates were from dogs (58.8%), 27 from cattle (39.7%), and one from a jackal (1%). The distribution and number of rabid animals by region are shown in Table 1.

**Table 1:** Rabid animals detected in Georgia in 2015–2016, by region, for which specimens were submitted for phylogenetic analysis.

**Region of Georgia**	**Source animals for viruses undergoing phylogenetic analysis (n)**		
	**Dog (** ***Canis lupus familiaris*** **)**	**Cattle (** ***Bos taurus*** **)**	**Jackal (** ***Canis aureus*** **)**
Abkhazia^*^	-	-	-
Adjara^*^	4	1	-
Guria	2	9	-
Imereti	11	4	-
Kakheti	6	4	-
Kvemo Kartli	5	1	-
Mtskheta-Mtianeti	-	1	-
Racha-Lechkhumi/Kvemo Svaneti	-	-	-
Samegrelo-Zemo Svaneti	4	7	-
Santskhe-Javakheti	-	-	-
Shida Kartli	4	-	-
Tbilisi	4	-	1
Total	40	27	1

^*^Denotes occupied territory or autonomous republic.

### 3.1 Real time RT-PCR, rapid typing, and phylogenetic analysis

**Figure 1:**
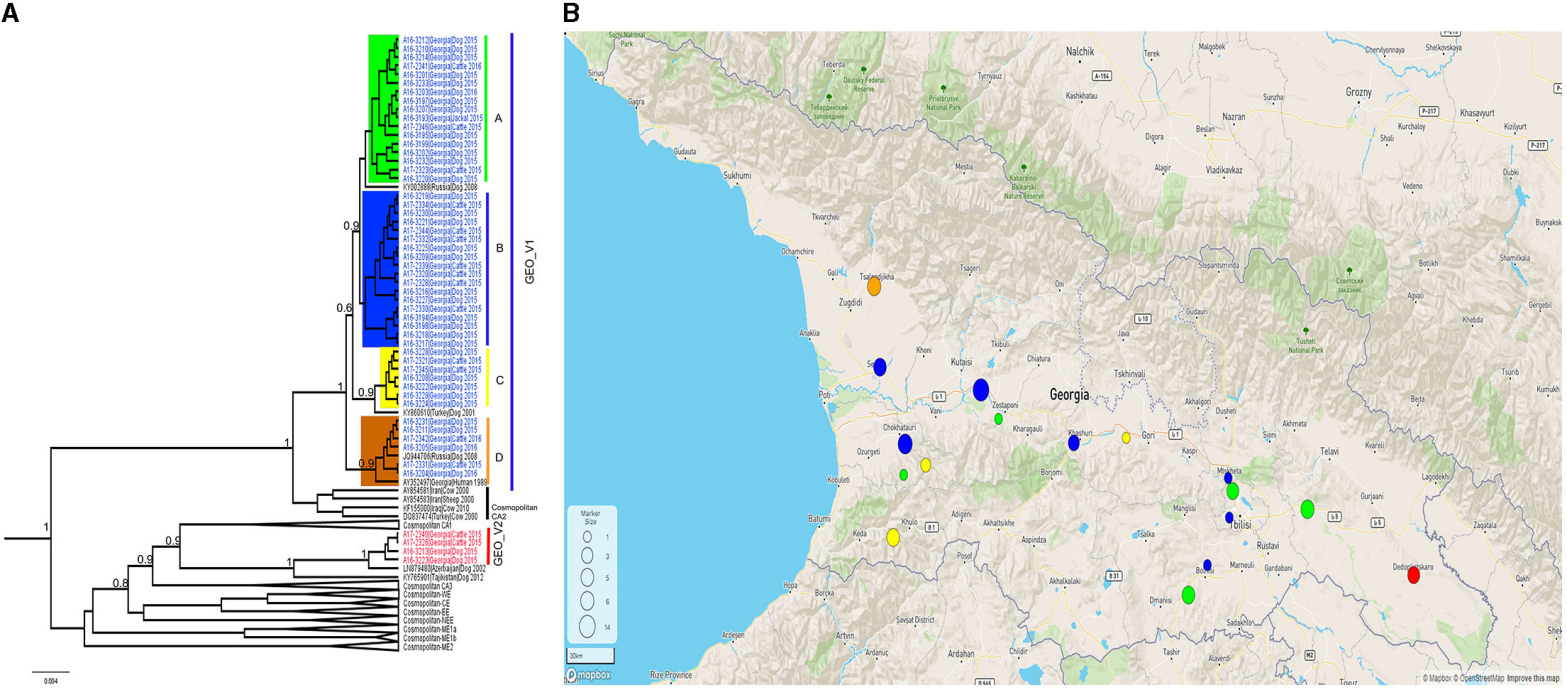
**(A)** A Bayesian phylogenetic tree of the complete N gene was reconstructed using 107 complete N gene sequences. The branches of Georgian phylogroups are outlined in different colors. The GEO_V1 in blue was dispersed across the country and further split into four sub-groups; only nodes with a posterior support value higher than 0.6 leading to the Georgian phylogroups are indicated in this figure. Nodes with posterior support <0.6 are not indicated. The number in the scale bar is the number of substitutions per site. **(B)** Map generated using Microreact. Dots outlined in Georgia's geopolitical map represents the number of samples collected and the colors show the phylogroup/sub-group to which they belong. Sample locations were randomly assigned to region level.

All the samples tested positive by the real-time RT-PCR LN34 assay. The Ct values for the LN34 assay and β-actin were below the cut off values (LN34 ≤ 35 and β-actin ≤ 33) (Supplementary Table 1). Phylogenetic analysis of the complete N gene was performed using a subset of 52 representative Georgian sequences and 55 representative sequences obtained from GenBank. The analysis distinguished two monophyletic phylogroups (GEO_V1 and GEO_V2) of RABVs in the phylogenetic tree (Figure 1). With well posterior support at the tree nodes, both Georgian groups were closely related to the cosmopolitan dog clade of rabies previously described in Central Asia (CA) (Troupin et al., 2016). Although both phylogroups included isolates from dogs and cattle, GEO_V1 grouped most isolates with a larger geographic coverage.

The phylogroup GEO_V1 was widely dispersed across the country and split into four sub-groups (A-D) that overlapped geographically. GEO_V1-A, highlighted in green, included samples collected in the southeastern regions of the country (Kvemo Kartli, Kakheti, and Tbilisi). This sub-group included mainly dog and cattle samples; furthermore, a jackal sample from Tbilisi and two dog samples from Guria and Imereti were grouped within this sub-group. GEO_V1-B was detected in the central-western part of the country (Imereti, Guria, Samegrelo-Zemo Svaneti, and Shida Kartli); three samples from the eastern regions (Tbilisi, Kvemo Kartli, and Mtskheta-mtianeti) were also clustered into this sub-group. Most samples collected in the Adjara region, which is located in the southwestern corner of the country, formed the sub-group GEO_V1-C; those samples are highlighted in yellow, and include two other samples from the neighboring region of Guria and one from Shida Kartli. In addition, GEO_V1-C was closely related to KY860610 RABV from Turkey. GEO_V1-D, highlighted in orange, was circumscribed to the northwest region of Samegrelo-Zemo Svaneti and included samples collected from dogs and cattle; this sub-group includes an RABV from Krasnodar (JQ944706). Phylogroup GEO_V2 in red was made up of four samples collected in the Kakheti region in the country's southeast; this phylogroup was closely related to the RABV LN879480 circulating in Azerbaijan. Across the entire N gene, a unique amino acid substitution at position 448 was the distinctive characteristic of GEO_V2.

**Figure 2:**
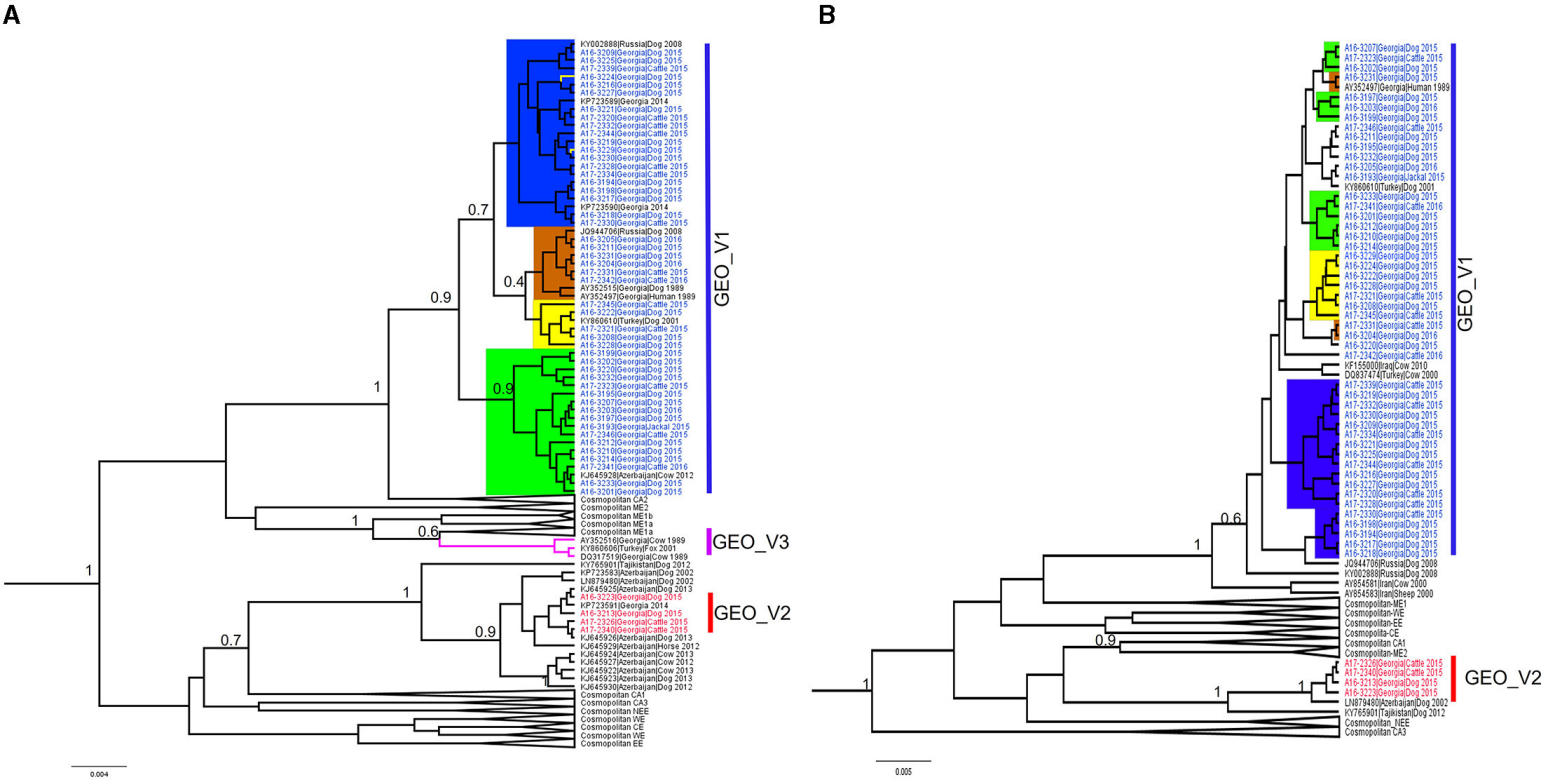
Bayesian phylogenetic analysis of Georgia isolates based on 400 bp. of the N-terminal and C-terminal of the nucleoprotein gene. **(A)** N-terminal phylogenetic tree, with phylogroups/sub-groups highlighted in different colors. Leaves labeled with blue in GEO_V1 were obtained in this study and other isolates from Turkey, Russia, and Azerbaijan were obtained from the public domain. GEO_V2 with red labels were part of this study; this phylogroup included isolates from Azerbaijan. GEO_V3 in purple was detected in the 1980s in Georgia and Turkey but not in this study. **(B)** C-terminal phylogenetic tree; blue- and red-labeled tips indicate sequences obtained in this study. In both phylogenetic trees, only nodes with a posterior support value higher than 0.6 that differentiate the Georgian phylogroups are indicated; nodes with posterior support <0.6 are not indicated. The number in the scale bar is the number of substitutions per site.

Most of the RABV sequences from the Caucasus region available in the public domain have a length of 400 bp at the N-terminal. We extended our data set to 124 sequences of the N-terminal to explore the relationship of the Georgian RABVs with those reported in neighboring countries. The topology of the phylogenetic tree produced by the N-terminal (Figure 2A) clearly distinguished phylogroups GEO_V1 and GEO_V2, and the majority of sub-groups were placed similarly to the complete N gene, however, two members (A16-3229 and A16-3224) that were part of the sub-group C in the complete N gene were placed into the sub-group B. Additionally, including partial N-terminal RABV sequences from Azerbaijan enlarged the phylogroup GEO_V2, and an isolate KJ645928 detected in Azerbaijan's region of Dashkesen was incorporated into the sub-group A. Overall, the phylogenetic tree produced by the N-terminal was comparable to the complete N gene in Figure 1A. However, sub-groups forming phylogroup GEO_V1, like those highlighted in yellow and orange, had a posterior support value below 0.6. Further, the phylogenetic analysis distinguished an additional phylogroup that we named GEO_V3, which is identified by the purple color in Figure 2A. In this study, we did not find any isolates closely related to the phylogroup GEO_V3 (DQ317519 and AY352516). The C-terminal phylogenetic tree, which included 107 sequences, separated both phylogroups similarly to the complete N gene in Figure 1A and N-terminal (Figure 2A); however, the posterior support for the sub-groups in the C-terminal phylogenetic tree was lower than 0.6, which can be observed in Figure 2B, where the resolution to differentiate sub-groups within the phylogroup GEO_V1 is limited.

**Figure 3:**
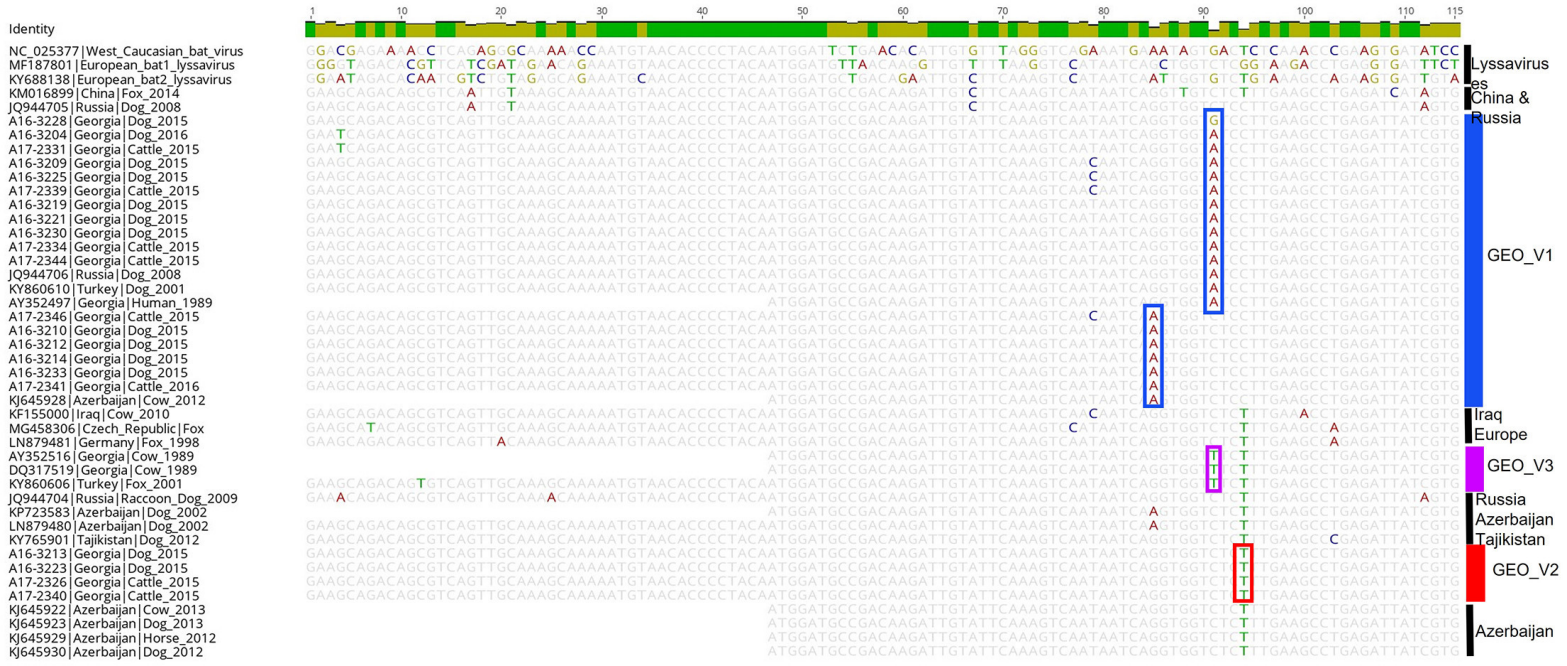
Shows the trimmed alignment (115 bp) of 41 RABV sequences and three other lyssaviruses. The length of the sequence corresponds to the amplicon obtained by the LN34 assay. Selected RABV samples from Georgia representing each phylogroup were chosen for pattern matches. The color on the side indicates the phylogroups determined by the complete and partial N gene analysis. Highlighted nucleotides in the alignment indicate the nucleotide changes that differentiate the phylogroups.

The qualitative results of the LN34 test were determined based on their Ct value (Supplementary Table 1). The LN34 amplicons (164 bp) were sequenced, and primer sequences were removed to conduct the alignment. Because of the many identical sequences of the LN34, only ≤ 5 identical sequences were considered for the sequence comparison. A subset of 44 representative sequences (115 bp) that includes Georgian samples and other lyssaviruses reference sequences were compared for pattern matches. GEO_V1 samples had unique patterns at positions 85 and 91 (Figure 3). Within GEO_V1, the sequences highlighted in green in Figure 1 had a single-nucleotide polymorphism (SNP) at position 85. A unique nucleotide pattern at position 95 differentiates the GEO_V2 from other closely related samples. The three sequences of GEO_V3 presented a unique nucleotide at position 91 that differentiated them from the previous two phylogroups.

## 4 Discussion

Past studies recognized that the Cosmopolitan dog clade of rabies could have been introduced into the Caucasus region and spread to Georgia in many ways, with some believing the introduction of the virus came from the Middle East and Europe (Horton et al., 2015). Over time, a small number of complete and partial N genes from Georgian RABVs sequences were used to conduct a phylogenetic analysis in different studies, but those studies left unanswered questions regarding their epizootiology. In this study, we analyzed the complete N gene sequence of the RABVs collected in Georgia during 2015 and 2016 and explored the link between lineages and their geographic distribution. Also, we analyzed the partial N gene (400 bp) from each end of the N gene. Further, we sequenced the amplicons of the LN34 assay that amplifies the highly conserved non-coding leader region and part of the N gene (Wadhwa et al., 2017; Gigante et al., 2018). The LN34 assay nucleotide pattern was compared to determine changes that lead to host differentiation and, on this basis, use the LN34 amplicons for rapid genetic typing.

All the sequences from Georgia obtained in this study were part of cosmopolitan lineages closely related to CA. The phylogenetic analysis revealed two distinct dog RABV phylogroups with a high posterior support (Figure 1). Most Georgian samples were clustered within phylogroup GEO_V1, which was closely related to the previously identified cosmopolitan CA2 sequences (Troupin et al., 2016). Geographically, this phylogroup was largely dispersed across the country, and further split into four sub-groups (A-D). Sub-groups A-C are clearly different from those clustered in sub-group D, and the posterior support can be guaranteed to be correct; however, the nodes that differentiate A, B, and C are slightly low (0.6), which indicates that the assertiveness of these sub-groups is low. This may have been caused by the number of informative sites in the data set (Simon, 2020). Four samples formed the phylogroup GEO_V2, which was restricted to the Kakheti region located in the southeast of the country; this phylogroup branched near the RABVs of the cosmopolitan CA1.

GEO_V1 consisted of sequences collected from dogs (*n* = 38), cattle (*n* = 25), and jackal (*n* = 1) that were closely related to the RABVs previously described as cosmopolitan CA2 which were detected in neighboring countries. Sub-group A, identified in green on the map, and the phylogenetic tree in Figure 1 is closely related to the strain KY002888 from the Dagestan region of Russia. KY860610 from Ardahan, Turkey, was found to be related to sub-group C, while strain JQ944706, previously found to be part of the cosmopolitan CA2 from the Krasnodar region of Russia, was found to be related to sub-group D. In addition, the partial N-terminal gene analysis incorporated the RABV from Azerbaijan (KJ645928) within GEO_V1. This inclusion revealed a wider dispersion of this phylogroup. It has been suggested that, besides the dogs, jackals, and wolves, there are also rabies reservoirs in Georgia (Imnadze et al., 2008). Our phylogenetic analysis showed that the RABV detected in a jackal was most likely due to a spillover of dog rabies because the sole isolate from a jackal collected in Tbilisi was clustered within sub-group A. Although the incidence of rabies in the wildlife of the Caucasus has been previously described, its magnitude remains unknown due to sample collection bias and is typically underreported (Vos et al., 2009; Zeynalova et al., 2015). Therefore, with limited data about rabies in wildlife, we cannot ensure that wildlife is maintaining an independent cycle of rabies in Georgia; only enhancing rabies surveillance on wildlife species will help to elucidate the rabies dynamics in jackal populations or other wildlife species.

A small number of samples from the Kakheti region formed the phylogroup GEO_V2. The complete N gene analysis showed that this phylogroup was closely related to the isolate found in Azerbaijan (LN879480) in 2002. Although GEO_V2 branched independently, genetically, this phylogroup was most closely related to the cosmopolitan CA1 RABVs that were found in Russia, Iran, and China. The extended data set for the partial N-terminal showed various isolates from Azerbaijan closely related to the GEO_V2, which indicates a wide transboundary dispersion. Previous studies suggested that the ancestor for the RABV KP723591 (Georgia) and others from Azerbaijan that were grouped into the GEO_V2 occurred in a wildlife population in Europe (Horton et al., 2015). Although reporting rabies cases in wildlife in the Caucasus is biased (Zeynalova et al., 2015), determining any rabies host-shift into wildlife with a limited number of confirmed rabies cases is difficult.

In addition, the N-terminal phylogenetic tree showed a few isolates that formed the phylogroup GEO_V3; those isolates were collected in the 1980s and early 2000s and were closely related to the cosmopolitan ME1a. In this study, we did not find any isolates closely related to those RABVs; therefore, further investigation is necessary to rule out if this phylogroup is persisting in Georgia or has been eliminated.

In developed countries, sequencing the whole genome has become a crucial tool for improving the public health system, but in low- and middle-income countries where rabies is endemic, implementing reliable molecular approaches to strengthen the laboratory capacity is often constrained due to limited resources (Brunker et al., 2020). Partial N gene sequences, particularly the first 405 bp of the N gene, provided suitable data to differentiate rabies genotypes (Kissi et al., 1995). Also, a recent study used a partial sequence of the end of the N gene (726 bp) to monitor rabies control programs (Binkley et al., 2021), and other studies in Southern Africa and India used the middle region of the N gene (Reddy et al., 2011; Muleya et al., 2019; Kainga et al., 2023). Therefore, there is no consensus on the length or direction of the sequence that is more reliable for conducting a phylogenetic analysis. Because most rabies sequences from the Caucasus region available were obtained in Azerbaijan, we examined the phylogenetic trees produced by the 400 bp length of the N-terminal and used the same length of the C-terminal (Figure 2). Both trees were able to differentiate the phylogroups GEO_V1 and GEO_V2. However, the confidence to differentiate the sub-groups was slightly reduced using the N-terminal of the N gene, particularly because the posterior support was low (0.7) and two isolates were internally rearranged, which caused a disagreement with regards to the complete N gene analysis. The disagreements were even more evident on the C-terminal phylogenetic tree, wherein the posterior support was low (not indicated in Figure 2) and impacted the resolution to distinguish the sub-groups. Although a previous study mentioned that the first 400 bp of the amino terminus of the N gene is highly variable, this helped to differentiate rabies isolates collected from different parts of the world (Kissi et al., 1995). In our study, the analysis of the partial N-terminal produced a tree narrowly different from the complete N gene, but the main Georgian phylogroups were clearly different from each other. Regarding the C-terminal, the internal sub-groups were incomparable to the complete N gene in Figure 1.

The LN34 sequences were used to rapidly determine the reservoir host and detect earlier rabies infections in surveillance samples (Gigante et al., 2018; Condori et al., 2020; Dettinger et al., 2022). In this study, a nucleotide sequence pattern comparison of the 115 bp length of the LN34 amplicons of Georgian samples (Figure 3) showed unique nucleotide differences between phylogroups GEO_V1 and GEO_V2 and other lyssaviruses. Although the sequence is short and the number of representative sequences from the Caucasus region publicly available is reduced, the analysis of LN34 sequences was capable of distinguishing the three phylogroups that circulated in Georgia. Therefore, this approach can be useful to conduct rapid genetic typing but not for phylogenetic analysis because the availability of sequences covering the LN34 fragment in the public domain may be limited and the sequence length is short. The advantage of sequencing the LN34 amplicons relies on its cost-effectiveness and short turnaround time. It also has improved sensitivities for the genetic typing of samples in poor condition or low viral loads; therefore, it may be considered a tool for genetic typing, especially in rabies-endemic countries where access to sequencing the complete genome is restricted.

## 5 Conclusions

Although the sample period is limited to 2 years, the aim of our study was to provide the initial data on molecular epidemiology and attempt to fill the current gaps in order to help implement effective control measures to protect human and animal health in Georgia. The presence of the Cosmopolitan dog clade, also known as the Central Asia (CA) clade, was confirmed in Georgia by sequencing the complete N gene. Furthermore, the phylogenetic analysis distinguishes two distinct phylogroups that are closely related to CA1 and CA2, and which overlap in many areas with transboundary dispersion. Partial N gene analysis of the N-terminal produced a tree slightly different from the complete N gene. Despite being less robust, analyzing the first 400 bp can still be suitable for epidemiological studies (Kissi et al., 1995). However, this should be taken with prudence when closely related samples are analyzed. The C-terminal analysis showed limitations in identifying sub-groups. The advantages of analyzing LN34-derived amplicons have been demonstrated to be a useful basic tool to differentiate the RABVs circulating in dogs in Georgia and can be used to monitor vaccination campaigns and determine the introduction of new rabies variants. Further, investigating the role of wildlife species as rabies reservoirs in the Caucasus region is clearly needed to fill the current gaps in molecular epidemiology.

## Data availability statement

The datasets presented in this study can be found in online repositories. The names of the repository/repositories and accession number(s) can be found below: https://www.ncbi.nlm.nih.gov/genbank/, MT079888 to MT079955.

## Ethics statement

Ethical approval was not required for the study involving animals in accordance with the local legislation and institutional requirements because samples of animals were collected as part of rabies surveillance in Georgia.

## Author contributions

RC, LN, and YL conceptualized the study. RC and AW performed real time RT-PCR. RC performed the standard PCR and sequencing, analyzed the data, and wrote the first draft of the manuscript. NK, LA, MD, TE, and GM collected epidemiological data. RC, NK, and YL validated the data. CM, LN, MR, EP, and YL revised the manuscript. All authors contributed to the article and approved the submitted version.
